# Small-scale environmental enrichment and exercise enhance learning and spatial memory of *Carassius auratus*, and increase cell proliferation in the telencephalon: an exploratory study

**DOI:** 10.1590/1414-431X20198026

**Published:** 2019-04-25

**Authors:** C.C. Abreu, T.N. Fernandes, E.P. Henrique, P.D.C. Pereira, S.B. Marques, S.L.S. Herdeiro, F.R.R. Oliveira, N.G.M. Magalhães, D.C. Anthony, M.A.D. Melo, C. Guerreiro-Diniz, D.G. Diniz, C.W. Picanço-Diniz

**Affiliations:** 1 Instituto de Ciências Biológicas, Hospital Universitário João de Barros Barreto, Laboratório de Neurodegeneração e Infecção, Universidade Federal do Pará, Belém, PA, Brasil; 2 Laboratório de Biologia Molecular e Neuroecologia, Instituto Federal de Educação Ciência e Tecnologia do Pará, Bragança, PA, Brasil; 3 University of Oxford, Department of Pharmacology, Mansfield Road, Oxford, United Kingdom

**Keywords:** Carassius auratus, Spatial learning and memory, Object recognition, Stereology, Telencephalon, Environmental enrichment

## Abstract

*Carassius auratus* is a teleost fish that has been largely used in behavioral studies. However, little is known about potential environmental influences on its performance of learning and memory tasks. Here, we investigated this question in *C. auratus*, and searched for potential correlation between exercise and visuospatial enrichment with the total number of telencephalic glia and neurons. To that end, males and females were housed for 183 days in either an enriched (EE) or impoverished environment (IE) aquarium. EE contained toys, natural plants, and a 12-hour/day water stream for voluntary exercise, whereas the IE had none of the above. A third plus-maze aquarium was used for spatial and object recognition tests. Different visual clues in 2 of its 4 arms were used to guide fish to reach the criteria to complete the task. The test consisted of 30 sessions and was concluded when each animal performed three consecutive correct choices or seven alternated, each ten trials. Learning rates revealed significant differences between EE and IE fish. The optical fractionator was used to estimate the total number of telencephalic cells that were stained with cresyl violet. On average, the total number of cells in the subjects from EE was higher than those from subjects maintained in IE (P*=*0.0202). We suggest that environmental enrichment significantly influenced goldfish spatial learning and memory abilities, and this may be associated with an increase in the total number of telencephalic cells.

## Introduction

The most primitive of the vertebrates, fish, have been described as possessing a poor differentiated telencephalon, and limited learning and memory abilities (1). However, developmental, neuroanatomical, and functional studies suggest that the evolution of brain and behavior systems seems to be conserved in all vertebrates (2,3), including teleost fishes (4,5), where we can recognize many of the learning and memory abilities shared by mammals (6) and birds (7).

Object (what?), timing (when?), and placement recognition are well-established cognitive functions in birds and mammals that have been associated with hippocampal neurogenesis and gliogenesis (8,9). Fish cell proliferation is affected by environmental changes but it has been suggested that fish may respond to the environment through processes that are not specific to behavioral change (10). Fish differ from birds and mammals (11,12), and thus regional cell-proliferation specific responses in fishes should be interpreted under rigorous control of environmental variables to guarantee specificity of the environmental stimuli (13) (see (10) for recent review).

A few studies have investigated potential influences of environmental enrichment and exercise on spatial learning and memory performances in teleost fishes (14,15) and none have investigated the effects of enriched environments on cell proliferation in the *Carassius auratus* telencephalon.

Although several *C. auratus* studies explored the remarkable parallelism between central nervous system morphology and functional organization of fishes, mammals, and birds (16–23), none of them investigated potential influences of environmental changes on learning and memory performances, and on telencephalic cell changes, using unbiased stereological methods. The present research addressed this question controlling for all other sources of potential confounding factors, including water temperature, pH, O_2_ concentration, day-night cycle, noise level, and sex and number of individuals per volume of water in the aquarium.

The telencephalon was selected as the area of interest for counting cells because it has been described in *C. auratus* that its integrity, either medial (24,25) or lateral (18 [Bibr B19] [Bibr B20] [Bibr B21],^22^,^23^,^26^–28), are essential for spatial learning and memory.

## Material and Methods

Female *C. auratus* were maintained in aquariums in accordance with the guidelines published by the National Institutes of Health (Guide for the Care and Use of Laboratory Animals). The experimental protocol was submitted and approved prior to study initiation by the Ethics Committee on Experimental Animal Research (Instituto de Ciências Biológicas, Universidade Federal do Pará, Brazil, CEPAE-UFPA: 0432015).

**Figure 1.**
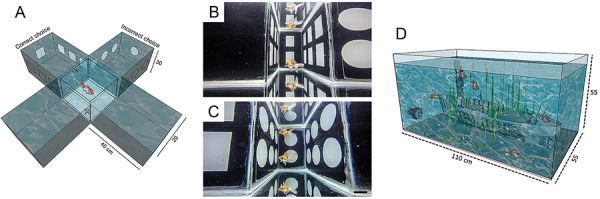
**A**, Illustration of the plus-maze apparatus. **B**, Correct choice in behavioral test. **C**, Incorrect choice in behavioral test. **D**, Illustration of enriched environment aquarium. Scale bar in **B** and **C**: 10 cm.

In the present research, we investigated the influence of small-scale environmental enrichment and exercise on spatial learning and memory in *C. auratus* using visual cued plus-maze apparatus (Figure 1A–C) and evaluated the changes in the number of cells of the telencephalon. We used an unbiased quantitative stereological method for cell counts, the optical fractionator (29). To that end, we maintained 6-month-old fish for 183 days (6 months) in two distinct aquariums (110×55×55 cm) with a capacity of 332 liters. All aquariums contained biological filters (Sunsun Jp-025f 1600 L/h, Japan), internal circulation pump (Sunsun Wave Maker jvp-102b 5000 L/h 110V), thermometers (Aquarium digital thermometer), stones, ultra violet lamp, and 12-h light/dark cycle. On alternate days, oxygen, pH, and temperature were measured and kept within the acceptable standards for this species (oxygen above 6.0 ppm, pH=7.0−7.6, and temperature 22±1°C). The mean values and standard errors were oxygen (EE: 6.95±1.8; AP: 7.21±1.5), pH (EE: 7.1± 0.09; IE: 6.7±0.8), and temperature 22.4±0.04°C). The fish were fed twice daily with commercial feed (Sera Pond Bio Granulat, Brazil).

The enriched environment aquarium was equipped with a water pump that generated water flow for voluntary exercise, natural plants, and a resin boat to provide resting and shelter (Figure 1D). The impoverished aquarium did not contain any of these devices.

### Memory and learning test

A plus-maze test apparatus (30) adapted to our experimental requirements (test aquarium) was used to assess spatial learning and memory performances. The plus-maze aquarium was maintained with similar water control parameters as previously described. Visual cues were placed on two arms of the apparatus, where squares indicated the right choice and circles the wrong one. The test was performed during three consecutive days; all fish performed 10 trials, reaching a total of 30 sessions by the end of the 3rd day, if the right choice did not occur before. All animals were adapted by entering into the apparatus 5 min before the beginning of the test. The trial started in the center of the apparatus, where fish remained for 1 min, inside a rectangular glass lid. Then, the glass was removed, and the individual had free access to the 4 arms of the apparatus. As soon as the correct choice was selected, the individual was removed from the test aquarium and returned to its original aquarium. If the wrong choice was selected, we imposed a 1-min restriction on the swimming space, using a containment net at the bottom corners of the chosen arm, and then fish were transferred to their original aquarium. This restriction was systematically applied to all fish until the criteria to complete the task were reached, or the 30 sessions ended.

After behavioral tests, all animals were sacrificed using an overdose of 20% Avertin (tribromoethanol amyl alcohol, Sigma-Aldrich, USA) dissolved in the water of a small aquarium.

### Statistical analysis of behavioral data

To assess learning rate and memory, we used Kaplan-Meier survival curve. A Kaplan-Meyer curve predicts time until an event and shows a series of declining horizontal steps, which provides, as a function of time, the true survival function for the sample under analysis. The main obstacle to the widespread use of survival analysis (such as in the present research) is the word “survival,” which may lead to the misunderstanding that it can only be used for data related to death or failure (40). In the present work, we have made the following question: How long does it take for a fish to learn and remember the correct arm? (time for the occurrence of the correct choice versus wrong choice). Here, the event was reaching the learning and memory criteria as a function of the progression of the training session (time). In our sample, every time an individual achieved three consecutive correct choices, or 7 correct choices alternated with wrong ones, in a total of 10 trials, a decrease in the survival curve occurred (curve stepdown). Thus, the Kaplan-Meier survival curve in the present work illustrated the probability of the correct choice being made as a function of the number of training sessions. Because the EE individuals met the criteria faster than those maintained in an IE, their Kaplan-Meier curve showed a lower survival rate. The log-rank test was used to compare the two curves for significant differences.

### Fixation and histological procedures

**Figure 2.**
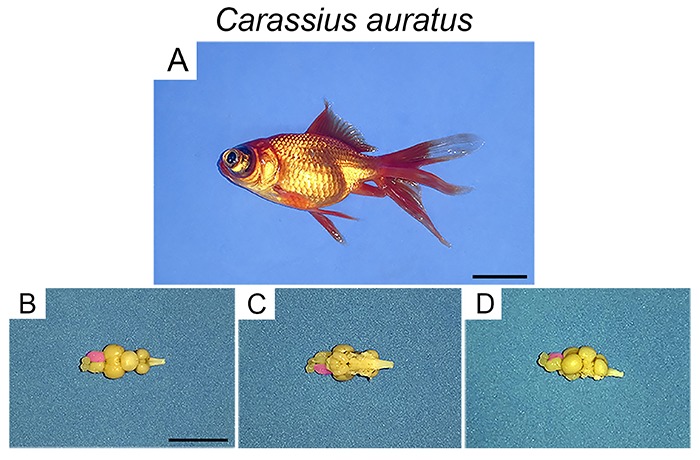
**A**, *Carassius auratus*. Dissected brain in dorsal view (**B**), ventral view (**C**), and lateral view (**D**). The right hemisphere is shown in pink. Scales: **A**: 2 cm; **B**, **C**, and **D**: 1 cm.

After craniotomy, brain tissue was fixed by immersion in buffered 10% formalin. Figure 2 illustrates a *C. auratus* specimen and its dissected brain in the dorsal, ventral, and lateral views. After 7 days, the brain was dissected and cut using a vibratome. Serial sections 70 µm thick were collected and 1:3 anatomical series were kept in the same fixative and maintained at 4°C in the refrigerator. They were then mounted in gelatinized glass slides, air-dried and stained with cresyl violet. After that, they were dehydrated, cleared, and mounted with DPX Mountant and cover-slipped (Sigma-Aldrich).

### Photomicrography

Digital photomicrographs were taken with a digital camera (Microfire, Optronics, USA) attached to a Nikon microscope (Optiphot-2, USA). The levels of brightness and contrast applied to the entire image were adjusted with Adobe Photoshop CC 2018 (USA).

### Stereology

To investigate the influence of the environment on the total telencephalic cell numbers (neurons and glia), we compared the stereological estimates of the total number of cell counts from 7 individuals of each group, using the optical fractionator (29). The stereological analysis requires the correct identification of the region of interest. The telencephalon of *C. auratus* shows, as all other teleost fishes so far investigated, two solid hemispheres composed of several nuclear masses separated by a common ventricle (31). The boundaries of the telencephalon are readily recognized in Nissl-stained sections of teleost fishes (31). To count cells, we used the systematic and random distribution of counting blocks in telencephalic parasagittal sections. This sampling is a key step since it is not possible to count all cells within the region of interest. To overcome this dilemma and obtain estimates close to the real values, the stereological procedure requires the use of systematic and random data collection. This alternative ensures adequate estimation of the total number of cells from the number of cells detected in each sampled counting box and in the sample probability (32). However, to minimize methodological errors we needed to select grid and counting box dimensions that generated, after counting procedures, a Scheaffer coefficient of error smaller than 0.05. Methodological errors ≤0.05 contribute little to the observed group variance. To fulfill this criterion, a pilot study was carried out where we tested different grid and counting boxes dimensions for the telencephalic sections of *C. auratus*, and counted cells, until we found appropriate coefficient of errors, increasing precision of the estimate.

## Results

All animals were weighed before sacrifice and had the same age at sacrifice. The two-tailed *t*-test to detect potential differences in the body weight of the two experimental groups did not show any significant difference (EE=22.04±2.49 *vs* IE=21.0±1.44, t=−0.98, P=0.35).

### Area of interest, grid, and counting boxes

**Figure 3.**
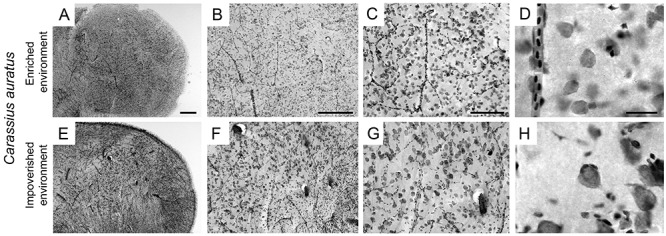
Photomicrographs of regions of interest (telencephalon) of *C. auratus* from enriched environment and impoverished environment, stained with cresyl violet dye. Magnification and bar size: **A** and **E**: 4x (500 μm), **B** and **F**: 10x (250 μm), **C** and **G**: 20x (250 μm), **D** and **H**: 100x (25 μm).

**Figure 4.**
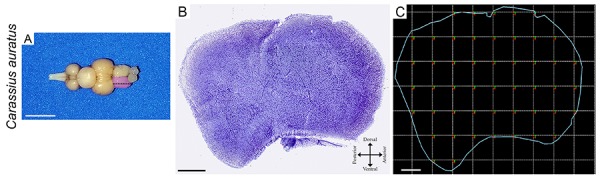
**A**, Photograph of the whole brain of *C. auratus*. The pink region indicates the right telencephalon, and the dotted line the location of the histological cut. **B**, Histological section of the telencephalon stained with cresyl violet showing how it was disposed on a slide. **C**, Outline of the systematic and random distribution of count boxes in the telencephalon region (blue line), demonstrating the layout and relative dimensions of the counting boxes (squares). The green edges of the count boxes demonstrate the limits of cell counting in the planes and the red edges the excluded plane limits. The width and length of the boxes is 30 μm and the grid (in dotted lines) is 320 μm. Scale bars in **A**: 5 mm; **B**: 400 μm; **C**: 320 μm.

Figure 3 shows low- and high-power photomicrographs of parasagittal Nissl-stained sections of the telencephalon of *C. auratus* maintained in enriched (A–D) and impoverished (E–H) environments. To count cells, we did not distinguish between glia and neurons. Figure 4 indicates the right telencephalic hemisphere, a parasagittal cresyl violet-stained section taken from the level illustrated by the dotted line over the pink hemisphere, and the systematic and random sampling approach for counting cells. Arrows under the stained section indicate anatomical references. Counting boxes are placed in the corner of each square of the chosen grid using Stereo Investigator software (MBF Bioscience, USA). The colored lines in counting boxes indicated prohibited (red) and allowed (green) counting lines.

Table 1 shows the stereological parameters selected to minimize potential methodological errors and Tables 2 and 3 show the estimates of the total number of glial and neuronal cells of *C. auratus* maintained in EE and IE aquariums. Compared with the total number of estimated cells on the telencephalon of fish maintained on the IE aquarium, on average, the total number of cells of individuals from EE was significantly higher (means±SD, EE=1.12×10^6^±0.23×10^6^ *vs* IE=0.86×10^6^±0.16×10^6^; two-tailed *t*-test, t=2.3, P=0.04).

**Table 1.** Stereological parameters for counting telencephalic cells of *C. auratus* maintained either in an environmentally enriched (EE) or impoverished (IE) aquarium.

*Carassius auratus*	Thickness (µm)	Volume (mm^3^)	Total of probes	Counting boxes total
EE 02	13.3	5.78	8	277
EE 03	18.3	4.14	7	199
EE 04	17.6	5.67	7	278
EE 05	16.3	5.50	8	266
EE 08	16.2	5.52	8	272
EE 11	14.3	7.32	8	359
EE 12	16.3	6.48	7	316
Mean	16.04	5.77	7.67	
SE		0.37		
IE 01	18.0	5.30	6	256
IE 02	16.0	8.93	8	431
IE 06	13.4	5.01	7	242
IE 07	14.0	6.40	8	315
IE 08	14.7	7.32	8	353
IE 04	16.2	5.02	7	242
IE 09	17.1	8.00	8	388
Mean	15.63	6.57	7.33	
SE		0.59		

Dimensions: box size 30x30 µm, grid 320x320 µm, dissector height 10 µm, and interval between sections 1:3.

**Table 2.** Estimation of the total number of glial and neuronal cells with their coefficient error for the *Carassius auratus* telencephalon of the enriched environment.

*Carassius auratus*	Total cells	Thickness (µm)	Scheaffer CE
EE 02	864,690	13.3	0.045
EE 03	995,356	18.3	0.060
EE 04	983,777	17.6	0.049
EE 05	1,103,967	16.3	0.048
EE 08	1,015,602	16.2	0.056
EE 11	1,380,236	14.3	0.045
EE 12	1,513,172	16.3	0.050
Mean	1,122,400	16.0	0.050
SD	235,477		
CV	0.2098		
CV^2^	0.044		
CE^2^	0.0025		
CE^2^/CV^2^	0.057		
CV^2^-CE^2^	0.04		
CVB^2^ (%)	91		

EE: enriched environment; CE: coefficient error; CV: coefficient of variation; CVB: coefficient of biological variation; CVB^2^=CV^2^-CE^2^.

**Table 3.** Estimation of the total number of glial and neuronal cells with their coefficient error for the *Carassius auratus* telencephalon of the impoverished environment.

*Carassius auratus*	Total cells	Thickness (µm)	Scheaffer CE
IE 01	723,446	18.0	0.057
IE 02	803,130	16.0	0.048
IE 06	708,942	13.4	0.052
IE 07	1,075,795	14.0	0.052
IE 08	1,096,997	14.7	0.045
IE 04	661,802	16.2	0.050
IE 09	975,856	17.1	0.045
Mean	863,709	15.6	0.050
SD	169,049		
CV	0.1957		
CV^2^	0.038		
CE^2^	0.0025		
CE^2^/CV^2^	0.06		
CV^2^-CE^2^	0.035		
CVB^2^(%)	92.1		

IE: impoverished environment; CE: coefficient error; CV: coefficient of variation; CVB: coefficient of biological variation; CVB^2^=CV^2^-CE^2^.

### Spatial learning and memory

**Figure 5.**
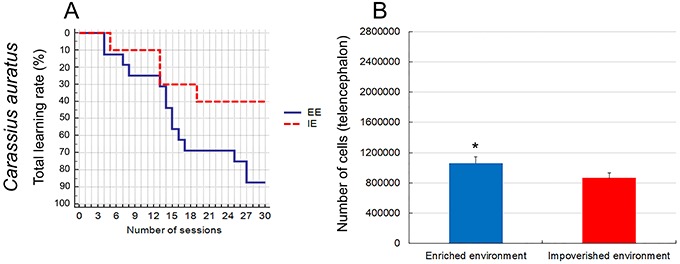
**A**, Graphical representation of the behavioral test of enriched (blue) and impoverished (red) groups of *C. auratus* (Kaplan-Meyer analysis, P=0.0354) in all animals. **B**, Estimated number of glial and neuronal cells in the telencephalon using the optical fractionator in the two experimental groups (P=0.0404). Data are reported as mean±SD. *P<0.05 (two-tailed *t*-test). IE: impoverished environment; EE: enriched environment.

Figure 5 is a graphic representation of the learning rates and the total number of telencephalic cells of the two groups. Compared to performances of fish maintained in the IE aquarium, subjects from the EE learned faster how to locate and remember the correct arm to be explored (log-rank test, P=0.0354). Compared with individuals from IE, the total number of telencephalic cells was significantly higher in fishes from EE (two-tailed *t*-test, P=0.04; see Tables 2 and 3 for details).

## Discussion

Evidence suggests that *C. auratus* can navigate on the basis of allocentric maps and this is dependent on the integrity of the telencephalon (18,22,23,26–28). In the present study, we found that compared with individuals maintained for 6 months in an IE aquarium, the total number of cells in the telencephalon of individuals maintained in an EE aquarium was increased, and this coincided with a better performance on the learning and memory plus-maze task. These findings are in line with previous evidence in mammals and birds demonstrating a significant increase of the total number of neurons and glial cells on the hippocampus of a variety species so far investigated (8,9).

Different from the endothermal mammals and birds, ectotherms do exhibit cell proliferation due to nonspecific influences of the environment, which may act indirectly through changes in body temperature (33,34), sex (35), age (36), and somatic and neural injuries followed by regeneration (37). These effects may influence cell counts (10). To minimize these influences, in the present study, we maintained experimental variables under control, including water temperature, pH, O_2_ concentration, day-night cycle, noise level, sex, age, and number of individuals per volume of water, in both environments. Thus, we expected that the significant differences found were rather specific.

As previously mentioned, the number of brain cells in teleost fishes increases with age, body weight, and body length throughout life, but studies on potential influences of environmental enrichment are scarce. Studies on telencephalic cell proliferation in the forebrain of zebrafish (*Danio rerio*) maintained in an enriched aquarium with artificial plants, demonstrated higher numbers of cells immunolabeled for proliferating cell nuclear antigen, suggesting that environmental changes may alter the cell cycle of zebrafish (38). Similarly, previous results in *Brachyhypopomus gauderio,* using bromodeoxyuridine as the cell marker, demonstrated an increase in cell proliferation across the brain in individuals maintained in a wild environment compared with the individuals maintained in captivity (13). Finally, in the Salmoniformes species *Oncorhynchus kisutch* and in the *Salmo salar* species subjected to environmental complex stimuli, an increase in BrdU (39) and NeuroD1 mRNA (15), respectively, was found in both dorsomedial and dorsolateral telencephalic regions compared with individuals maintained in a simple environmental structure.

Although we did not investigate the subjacent mechanisms in the present study, we expanded previous observations to *C. auratus*, demonstrating that the cell cycle of its telencephalon was also altered by small-scale physical environmental enrichment, and that these changes coincided with an enhanced performance on spatial learning and memory.

To quantify changes in telencephalic number of cells, we applied the optical fractionator, an accurate method of quantification combining properties of an optical dissector and the fractionator that has been used in a variety of studies to determine cell numbers in multiple brain regions. The optical fractionator is unaffected by histological changes or shrinkage, an issue of importance when performing comparative analysis between experimental groups (37,39,40). The main variability in the present analysis was biological variability (91–92% of the total variation), with CE/CV<0.5. As a result, possible variations associated with non-biological sources were reduced to acceptable levels.
